# Color-Patterns to Architecture Conversion through Conditional Generative Adversarial Networks

**DOI:** 10.3390/biomimetics6010016

**Published:** 2021-02-17

**Authors:** Diego Navarro-Mateu, Oriol Carrasco, Pedro Cortes Nieves

**Affiliations:** 1 School of Architecture, Universitat Internacional de Catalunya, 08017 Barcelona, Spain; 2 Institute for Advanced Architecture of Catalonia, 08005 Barcelona, Spain; oriol.carrasco@iaac.net; 3 Independent Researcher, 08018 Barcelona, Spain; pedro@pedrocortes.net

**Keywords:** machine learning, neuronal networks, cGANs, architecture, patterns, artificial intelligence, generative

## Abstract

Often an apparent complex reality can be extrapolated into certain patterns that in turn are evidenced in natural behaviors (whether biological, chemical or physical). The Architecture Design field has manifested these patterns as a conscious (inspired designs) or unconscious manner (emerging organizations). If such patterns exist and can be recognized, can we therefore use them as genotypic DNA? Can we be capable of generating a phenotypic architecture that is manifestly more complex than the original pattern? Recent developments in the field of Evo-Devo around gene regulators patterns or the explosive development of Machine Learning tools could be combined to set the basis for developing new, disruptive workflows for both design and analysis. This study will test the feasibility of using conditional Generative Adversarial Networks (cGANs) as a tool for coding architecture into color pattern-based images and translating them into 2D architectural representations. A series of scaled tests are performed to check the feasibility of the hypothesis. A second test assesses the flexibility of the trained neural networks against cases outside the database.

## 1. Introduction

Architects have a long tradition of using biomimicry, not only to bring coherence to design but also as a source of inspiration for problem solving. Natural systems offer strategies that improve performance and effectiveness in a wide formal repertoire and can be applied at different stages of the design process [1,2,3].

The biomimetic aspects of this research are manifested in two parts, always serving as analogies and methodologies that improve the application of computation for architecture. In other words, authors try to mimic biologic strategies to simulate approaches to a primitive, digitized architecture (proto-architecture).

The first biomimetic part revolves around the idea of DNA as a code, suggesting the possibility to encode tridimensional architecture representation into a very simple system like color patterns. The relevance of this approach is accepting that a code of extreme simplicity like DNA can be rearranged in ways that produce truly complex phenotypes, seamlessly solving hundreds if not thousands of geometric relations.

Authors propose to reinforce this relation between extremely simple coding and complex spatial phenotypes, embracing the idea that the connection is not fully understood but is still capable of generating valid results. An idea that leads to the concept of emergence and the opacity of neural networks [4,5].

Therefore, the main objective of the research is to test the capability of generative adversarial networks (GANs) to unfold color patterns in fully developed geometries that contain architectural elements and properties. In this conversion, special emphasis is placed on a non-direct transformation of information that could lead to neural networks capable of producing multi-scale designs from simple patterns.

The second biomimetic aspect builds on previous research, when the authors highlighted the tendency of geometric gradients to which most parametric modeling is anchored. While exciting, the digital momentum that has transformed the architecture thanks to algorithm aided design [6] has failed to incorporate all the flexibility that current biological models suggest. Thus, although evolutionary computation has acquired an important role in optimizing the field [7], the latest advances in evo-devo have not been incorporated into these algorithms, missing the opportunity to improve generative tools.

The integration of embryological processes based on gene regulation (patterns, body plans, enhancers, switches…) dramatically increases the modeling capability, generating great variety in their populations using light and efficient algorithms [8]. As will be seen, the generation capability is fundamental to the creation of a simple code (pattern) with the potential for emergence.

The paper is organized as follows. Section 1 is split in two subchapters: the first one describes the theoretical background, focusing on generative adversarial networks and their current applications. The second one relates patterns behavior and evo-devo regulation as a generative tool, considering data as the code to be compressed within the patterns, capable of decompress into architectural phenotypes. Section 2 describes the necessary tools and tests run in this research; it is also described the bio-parametric analogy in the evo-devo based algorithm and color-data system. Section 3 presents the results from the neural network and analyzes the data generated. Finally, Section 4 puts into perspective and concludes the ability of the neural network to corroborate the initial hypothesis.

### 1.1. The Relevance of AI and Its Impact in Recent Years

#### 1.1.1. Applications of Different Types of Machine Learning

From its inception in the late 1940s and since Alan Turing proposed the test [9] to measure machine intelligence and John McCarthy coined the term [10], Machine Learning (ML) has had a constant evolution toward the idea of the artificial brain. The field has experienced a late resurgence within the last decade, as a result of more accessible tools and hardware, set to solve traditional problems such as reasoning, representation, planning, learning and recognition [11]. One of the most advantageous abilities of these algorithms is the capacity to recreate specific tasks without the need to use explicit instructions [12].

Machine Learning methods like Deep Learning Neural Networks [13], Deep Belief Networks (DBN) [14], Recurrent Neural Networks (RNN) [15] and Convolutional Neural Networks (CNN) [16] have found applications in the field of computer vision, audio/speech recognition, machine translation, social network filtering, bioinformatics, drug design and much more.

Some of these algorithms rely on image databases and different training methods. Neural networks (NN) can learn and guess the contents of an image, like the one used in the experimentation of this paper. As a consequence, industries with large sets of data accumulated are more prone to use these algorithms. ML algorithms can be used in the healthcare industry to detect and assess health issues, where risks and threats can be predicted by finding patterns according to symptoms and genetic information in a patient’s medical history.

Artificial NN is already present in a multitude of other existing fields and is often used to predict human behavior and decision-making. E-commerce platforms already use them to find similar items to attract customers and streaming services use complex algorithms to predict users’ future interests.

Thus, in the short term, AI derivatives already represent an engine of change in the labor field that has and will have economic and political consequences (ref. the evolving nature of work).

#### 1.1.2. Generative Adversarial Networks

**Scheme 1:**
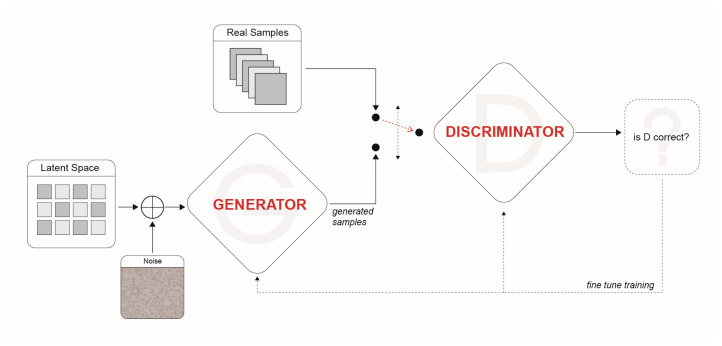
The structure of a Generative Adversarial Network (GAN) [21].

Within NN [17], Generative Adversarial Networks (GANs), first introduced by [18] are a set of two NN, one being the generator, with the ability to create new synthetic instances of data and the other the discriminator (Scheme 1). Within the generative models, adversarial networks have been some of the most successful ones but despite that, GANs are very difficult to train [19]. These NN have achieved remarkable performance on various tasks but suffer from training instability because the gradients given by the discriminator contain considerable adversarial noise, which, as a result, misleads the update of the generator and leads to unstable training [20].

Generative models need to be based on a function that is trained by a large set of data; that establishes the ground truth of the generator. The function will accordingly be able to generate a result related to the mentioned database. These networks can be used as generative models, capable of populating new data from a previously trained NN. Consequently, GANs have the possibility to generate non-existing data from the features that exist within the first data set. The other NN, the discriminator, classifies the data and correlates the output with the input, trying to filter and classify the real data and the falsely generated data [22].

#### 1.1.3. Conditional GAN

In the framework of this paper, a supervised conditional GAN is used as a solution to image-to-image problems, predicting pixels from pixels [23], using the pix2pix toolset. Later, Wang et al. [24] found a way to increase the resolution of the images, allowing for the use of higher resolution images, thus reducing the noise created by the generator and thus favoring the task of the discriminator.

**Scheme 2:**
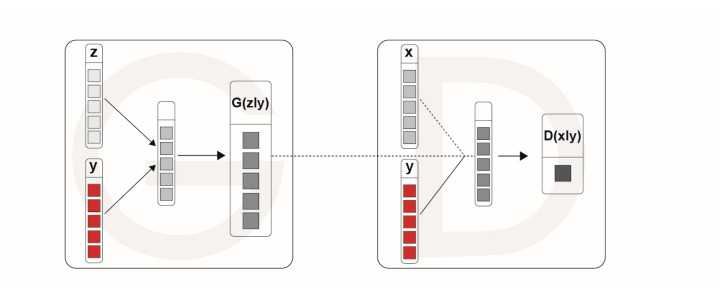
A condition dataset in a conditional Generative Adversarial Netweork (cGaN) [25].

The conditional in GAN models derives from the need to force the training in one direction [25] to avoid a certain lack of control on modes of the generated data. This can be achieved by feeding data that needs to be a condition on both the generator and discriminator. In the case of this paper, it is by using paired images for evaluating the results (Scheme 2).

#### 1.1.4. Machine Learning and Architecture

For the last two decades, architecture design has been deeply influenced by digital tools and by a digitalization process resulting from the implementation of building information modeling (BIM) methodologies (Autodesk Revit, 2000). The Parametricism style wave [26] allowed architects to explore shapes and forms that do not only rely on form or function in 1929 [27] but are a result of form-generating processes within. Architecture design concepts started to be much more closely aligned to form-driven design and performance-driven [28] as a result of digital form-finding methodologies.

Tools and a contemporary approach to architecture allowed for breaking the classical understanding of spaces, structures and form. Architecture is but can now be expressed as, the result of complex generative rules. These rules can be described as a pseudo-genetic language that can produce a code-script of instructions for form-generation [29]. The use of parametric code as a genetic code leads also to a morphogenesis process that allows the designer to establish a fitness criterion and appraise the evolution of the architecture itself [30].

Previous to the use of AI, ML and regardless of the use of BIM methodologies, more mundane approaches via heuristic methods [31] to generate automatic architecture plan distributions have been examined, all of them prior to the introduction of Grasshopper (Rutten, 2007), a tool that allowed the architectural industry to access algorithmic and generative modelling with ease. Physically-based planning [32], Constraint-based [33], Generative Design [34], Discursive Grammar [35], Heuristic Algorithms [36] and Genetic approaches [37,38] were explored among others methods.

**Figure 1:**
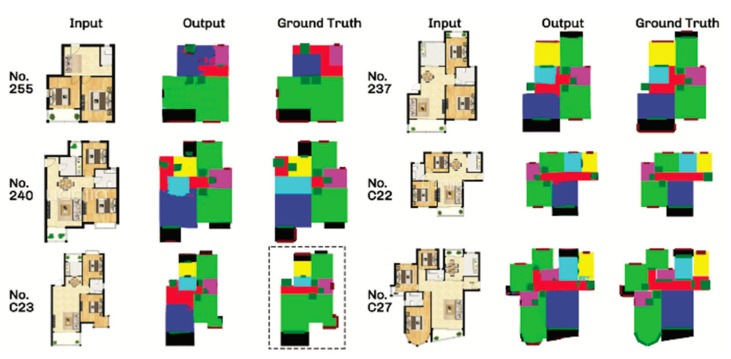
Floorplans training set [40].

The use of ML algorithms and NN has allowed adding more complexity and case studies to the topic of computer-generated architecture. Relevant recent works in this category worth mentioning are explorations from Stanislas Chaillou [39], using the proven creativity abilities of GaNs to generate different realities of the drafting process, all applied to floor plan designs. Within the same line of work, Huang & Zheng [40] propose codification for architectural elements (Figure 1), allowing a smoother learning experience for the NN, based on previous works done by Zheng et al. (2017) for generating urban and city-scale planning. Other examples from Mohammad et al. [41] on applying GaNs to design façades explore the relationship and symbiosis between architects and AI for making design decisions, which have proven to be successful.

In the current state, works and case studies involving architecture and ML algorithms tend to be primarily on the 2D representational space. Although proven powerful, more research in this field is needed in relation to a NN to comprehend the depth of objects for a 3D object reconstruction using convolutional architectures. Wu et al. [42] already have demonstrated the possibilities of bridging the gap via the so-called 2.5D to generate shapes and forms, based on previous studies about the prediction of 3D shapes [43,44,45,46].

### 1.2. Pattern Images as Genotypic Data for Architecture in Gans

#### 1.2.1. Patterns

From a generative point of view, it is absolutely essential to be able to encode information. Finding a way to mimic biological strategies to extrapolate their level of complexity to architecture will help to take advantage of the full potential of our computational tools. Information patterns can store and establish relations in data, can be scale aware while having fractal properties or adaptive while following a very limited set of rules [47].

The archetype of these processes is DNA. In the very same way that nucleoids compress the genomic data of DNA in a light and readable way, the computation could store architectural geometry inside within its series of bits, tweaking the weights within the NN [48].

DNA, also referred to as genetic code, clearly has similarities with mechanical or digital codes such as Morse, Baudot or ASCII, where long strings of information (nucleoid or binary) are converted into higher layers of information or symbols. As Richard Dawkins states: “the machine code of the genes is uncannily computer-like” [49].

In biology, ribosomes receive instructions from RNA to produce specific amino acids that will end up generating certain proteins. In our experiment, the parametric model receives the instructions from the color channels and converts them into geometrical commands that end up generating the architecture.

Among different types of data, digital images might be one of the most extended and useful ones. Authors have already exploited the characteristics within the internal structure of images in previous articles [50], making use of the channels associated with each pixel to convert data and express geometric relations among their pixels.

Bypassing the well-known world of CGI, the last few years have awakened a whole field like Computer Vision primarily focused on analysis [51] but also generating outcomes as has been mentioned in the GAN’s chapter. As one of the principal information channels, Images have established a strong relationship with the latest developments in AI.

Moreover, images can be the result of extremely complex processes, outcomes that can be understood as phenotypes, where astonishing geometry emerges through the combination of simple rules. Some well-known examples of this behavior are Alan Turing’s theory of morphogenesis based on reaction-diffusion chemical patterns [52], Lindenmayer’s tree branched L-Systems [53], Stephen Wolfram’s computing experiments for natural patterns generation [54], the genetic explanation of Evolutionary Development by Sean Carroll [55] or the “natural” cities of Steven Johnson [56]. No matter the field, patterns express the hidden relations within data.

It is without question that natural patterns can be useful to architecture in a wide range of usage and scales [57]. Material design [58], structure and façades [59] and the urban level [60] can follow many of the biologic rules that can be “easily” simulated through software [61].

These generations of patterns may lead to emergent and unexpected results to articulate a computational architecture that can be exponentially more powerful than contemporary architecture; an architecture that is more related to biologic and natural processes, working like a custom, adaptive and complex system focused on efficiency rather than optimization and standardization.

#### 1.2.2. Data Relation and Generation

Many GANs have proved resourceful when generating images from simplified images; the neural networks successfully identify shapes and are able to solve the relation between them; for example, GauGAN’s landscape generator [62] or the façade image translation from pix2pix-tensorflow [23]. An exploding field like self-driving cars is currently based on computer vision and tagging, which has led to datasets focused on object identification [51,63].

**Figure 2:**
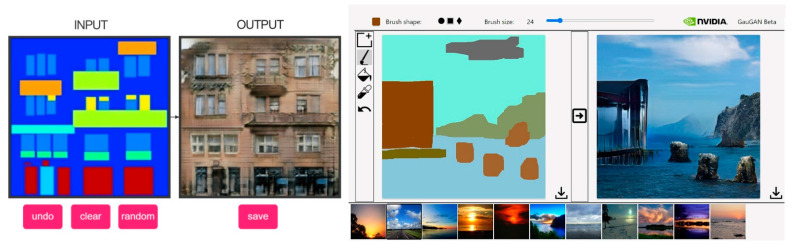
pix2pix (**left**) and Nvidia’s GauGAN (**right**). Screenshot showing the direct conversion from input and output.

However, all these attempts seem to keep a fairly direct relationship between the input layer (with ID colors) and the realistic output generated as can be seen in Figure 2. We could say that data has been simplified but the spatial relations inside the pixels have not changed. In the same way, there is no change in the typology of the image.

A truly light and complex code needs to break this direct relation to further simplify the information. If we refer back to the DNA example, no one could imagine that an endless string of four different letters could be converted into a full living being.

This research argues if it is possible that GANs understand coded patterns related to an architectural representation with images that are not directly related. How far can a GAN encode hierarchical changes in the structure of the data? How much can the data be simplified? How flexible is its understanding of cases outside the training?

Can a bi-dimensional pattern like a colored square grid become an isometric representation with architectural values?

#### 1.2.3. Parametric Design to Service Machine Learning

Unlike other fields, design jobs like architecture might have an advantage in introducing ML workflows into the discipline. Thanks to parametric and generative CAD software [6], architects can easily build and prepare their own database for NN training.

Any simple script is able to generate several million outputs [64] that can serve as image references for the NN. On top of that, the authors of this article have developed in previous research data workflows based on evolutionary-developmental biology in order to optimize performance and maximize variation [8]. These workflows have been applied to the current case study and will be better explained in the following chapter.

Moreover, the data used to generate those outputs is explicit in the script and can also be easily extracted and associated with the outputs, as well as converted in any necessary data type (like a pattern image). In other words, parametric designs can produce infinite examples with their input-output relations (genotype-phenotype conversion) to train NN models.

To exemplify the relevance of having access to the relationship between input and output, the authors point out that a large part of the development of AI today comes precisely to resolve or omit this relationship, either by using AI to find patterns [65] or in training NN that do not need to establish relationships between inputs and outputs [66].

## 2. Materials and Methods

### 2.1. Tools and Systems

Compared to other deterministic models, the use of deep learning offers unprecedented flexibility in the field of computing. However, the search for the perfect relationship between the different nodes of the NN also produces the adverse result of opacity.

The lack of understanding about what happens within the NN is not exempt from criticism [67,68] and everything seems to indicate that it will be necessary to incorporate measures to guarantee the correct functioning of the darker part [69].

To counteract this indetermination, a series of tests are developed to confirm the different parts of the hypothesis.

The first part of the experiment also has rendering and visualization implications. There are significant contributions to the research field of real-time rendering through the use of NN [70], even applied to the specific field of architecture [71]. However, they mainly focus on the direct conversion of geometry into materials and light simulation.

The work of Wu et al. [72] in Learning Shape Priors for Single-View 3D Completion and Reconstruction can be considered as a step further, where images with 3D information (normals, depth) are translated into 3D geometries.

As mentioned previously, one of the critical objectives of this experiment is to test the ability of a NN to convert relatively simple patterns into rather complex architectural spaces. Therefore, making the leap between the two-dimensional and the isometric representation is a fundamental part of the original hypothesis. In addition to the isometric representation, several rendering values have been added to the image, such as omni light and ambient occlusion.

In general terms, the case study consists in the generation of color patterns and architectural isometrics that are related through parametric rules. The designed algorithm, builds both inputs (patterns and isometrics) from an established set of relationships. These inputs are then used to train the neural network, in the hope that it will be able to “discover” and learn these established relationships. Throughout the experiment, successive configurations are adjusted to assess the learning of the neural network and the quality of the outputs.

#### 2.1.1. Software and Hardware

The experiments carried out in this research have been developed in Google Colab, a free tool from the Google ecosystem specific to the development of ML and deep learning applications. Colab allows remote use of Google GPUs and TPUs, although, as it is a free tool, Google limits the maximum connection time to its virtual machines, making it impossible to run more than twelve hours. The amount of memory available for the development is not constant in each session but it oscillates around 13 GB of RAM and 110 GB of Hard Drive.

The Colab development environment is defined in Python 3.6 (v3.6.x, 2019, USA) and has pre-installed many of the most-used libraries to develop ML applications. Among those that have been used for this research, we find:

- TensorFlow v 2.3.1
- Keras v 2.3.1.
- Scikit-learn v 0.22.2.post1
- Matplotlib v 3.2.2
- Numpy v 1.19.4

Google Colab specs (free version):

- CPU: Intel(R) Xeon(R) CPU @ 2.20GHz
- GPU: Tesla K80
- RAM: 12 GB

Test C03 (described later on) was run on a laptop with the following specs:

- CPU: Core i7-7700HQ de Intel (MSI Apache)
- GPU: GeForce GTX 1050 Ti de 4 GB
- RAM: 16 GB (DDR4)
- HD: 256 GB SSD

#### 2.1.2. Evo- Devo Modelling and Color-Data Systems

The parametric definition used follows prior research where a workflow founded on flow-based programming, polygonal meshes modelling and evo-devo strategies were established to maximize variation and efficiency [8].

Unlike the majority of current visual-programming architecture, adding the evo-devo capabilities encourages a truly emergent result. While the former tends to generate gradients and similar versions, reorganizing data as if it were genes allows the model to change more flexibly and freely.

The 1995 Nobel Prize in biology [73] raised a better understanding of how genes relate to the embryology development, evidencing a geometric and pattern-based structure within living beings. Body plans, allometric growths, genetic switches, homeobox genes and mutations describe a powerful tool kit based on hierarchies and data transformation [55].

**Figure 3:**
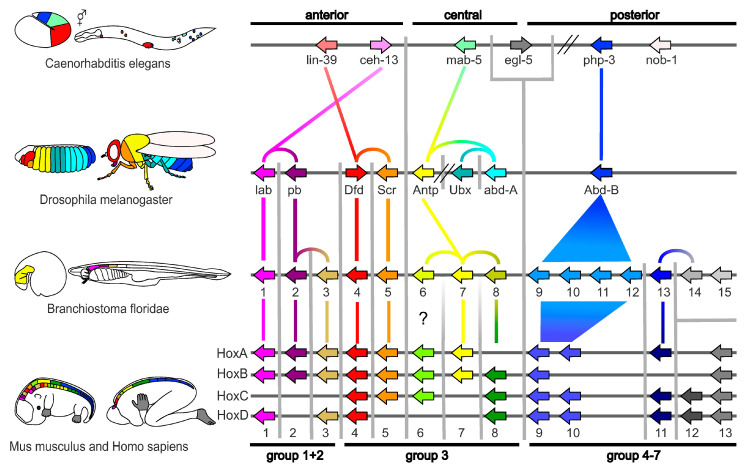
Scheme showing how homologous hox genes control embryonic development and hence the form of adult bodies [77].

Examples of these regulatory switches stablishing strong relations between biology and geometry can be found in Barbier et al., controlling spatiotemporal pattern formations [74]; or evolutionary enhancers studies in Drsophila from Matthew et al. [75] (Figure 3).

The digital architecture generated for this research makes use of a simplified version of the one developed by the authors but still faithful to the logic that prevails both in evo-devo biology and computation [76].

**Figure 4:**
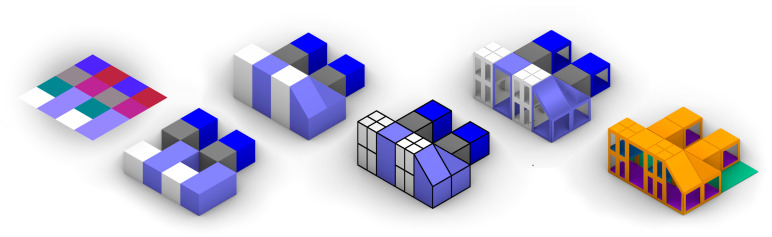
Evolution of the evo-devo model. From left to right: color-pattern (genotype), cells creation based on luminance, allometry (heights) based on red channel, subdivision (tiles) based on green channel, homeotic genes (openings) based on blue channel and final geometry (phenotype).

The generative algorithm is based on a bi-dimensional grid (4 × 4) populated by the color pattern that regulates the different modifications depending on the color (Figure 4). Following evo-devo mimetics, this data is explicit throughout the model; always latent inside the geometry-affecting different aspects of the architecture, turning on and off mesh transformations related to allometry (height), subdivision (tiles) and homeoboxes (openings). The result is an endless variation of architectural typologies.

Color-transformation association can be seen further on in Table 1.

As a data encoding experiment, it is important to maintain a balance between a simple and yet powerful system to generate diversity. This requires structuring the colors to associate them with possible phenotypes so that they all manifest themselves to a greater or lesser extent.

As already mentioned, the color system is closely linked to the generation of diversity that mimics the evo-devo processes. Colors work as genetic switches that activate different aspects of the parametric genotype; that is, meshes colors function as the patterns that trigger specific actions.

In this sense, color space science and the channels (RGB—Red, Green, Blue) that make up the colors are used as data paths but other factors such as luminosity (AHSL— Alpha, Hue, Saturation, Lightness) are also analyzed in order to expand the possibilities of the system [50,78,79].

Every channel in Table 1 is assigned to a specific transformation, where the specific lightness of the channel is analyzed. If the color is dark, the event does not trigger; if it is light, it does. The proportions between dark and light have been changed and are detailed in the Data Color Systems description.

#### 2.1.3. Data Color System 1

The first event is to create/delete cells within a rectangular grid. If the color is luminance and is below 0.5 (0–1), it gets deleted; if it is higher, a cubic cell appears.

After the cells have been generated, three transformations are applied based on the colors’ cell channels (RGB). The luminance of each channel is evaluated, and, again, depending on if it is lower or higher than 0.5, an event is triggered.

Color generation was restricted to specific ranges to facilitate their differentiation. However, the color distribution tends to create rather compact architectures with few empty spaces.

Early tests showed that increasing the number of color levels brought the ratio of created-deleted cells closer to 50%, which was more appropriate from an architectural point of view (Table 2). This behavior is due to the resulting luminance of the colors, which is not necessarily the mathematical addition of the RGB channel.

However, adding more levels of colors without directly associating them with genes expressing changes showed that the NN behaved less efficiently. For this reason, a new system was established, which may seem more complex as a starting point but which later turns out to be more direct and better associated with the parametric model and its characteristics.

#### 2.1.4. Data Color System 2

The second system is based on thirds shares (Table 3). It allows having the minimum number of colors (each one related to a specific modification/phenotype) while keeping a good balance in the proportion of events, including the creation/deletion cell.

As the diagram shows (Figure 4), instead of using a straight separation between dark and light to remove cells, a ⅓ conditional was used. This will produce an uneven distribution that is not harmful to the tests.

Each of the channels was divided into three levels, with the middle one slightly below the middle value. Because of this, averaging in that level results in 0 value.

As a result, there are 27 different colors with the following distribution of events:

### 2.2. Test Settings and Training

Fixed settings for all experiments in Table 4 unless it is specifically mentioned.

- Images used have been specifically dimensioned to 250 by 250 pixels.
- Output images set to 10.
- Ratio for generator-discriminator set to 80–20%.

The following tests progressively increase the complexity of the geometries as they require more complex coding systems through the color channels. Tests have been classified into four main objectives:

1. Group A of the tests is aimed at checking the hypothesis and assessing the impact of the settings on the algorithm.
2. Group B’s objective is testing the response to the increasing complexity.
3. Group C tests propose a different approach based on the pixel’s relations.
4. Group D checks the flexibility of networks trained with external inputs.

Two different types of geometries will be tested:

5. Cubic volumes (group A).
6. Architectural volumes (group B, C and D).

#### 2.2.1. Test A0#—Binary Patterns

**Figure 5:**
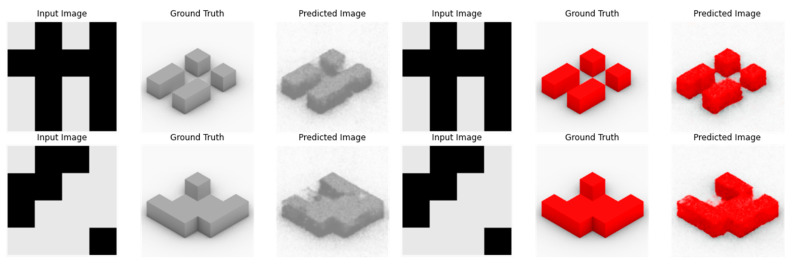
Outputs from A01 (gray) and A02 (red) Examples of the output generated by the first test. From left to right: pattern, expected result and generated image. Red isometrics better represent the “ground truth”.

To check the feasibility of the initial hypothesis and the ability to transform images in a non-linear way, the first test considers the conversion of binary patterns (black/white void/fill) into simple isometric volumes (Figure 5).

These tests are also aimed at having a better understanding of the settings and their implications.

- Test A01 (250-50) considers the simplest and initial case of conversion into grey volumes.
- Test A02 (250-50) compares the relevance of color by introducing red volumes that might help to distinguish between shadows and geometry.
- Test A03 (500-50) checks the impact of the database size.
- Test A04 (250-100) checks the impact of the number of epochs.

#### 2.2.2. Test B0#—Color Patterns

B-tests Introduce for the first time 27-colour patterns. As explained in previous chapters, each of the colors is split into channels to activate different events. To better analyze the results, the different objects of the architectural models are also colored: yellow-façade, Turkish-garden, purple-ground floor, blue-first floor.

- Test B01 (250-50) introduces for the first-time color patterns.
- Test B02 (350-70) increases the size of the database.

#### 2.2.3. Test C0#—Isometric Patterns

Due to the results in Group B, a third group (C##) of tests was developed where the initial conditions of the input image were changed from a square grid to an isometric representation of that same grid.

With this group of tests, the researchers look for a more efficient way to train the NN while testing the impact of more direct relations between the pixels in both images.

- Test C01 (250-50) introduces isometric patterns. To be compared with B01.
- Test C02 (350-70) increases database size. To be compared with B02.
- Test C03 (500-100) developed in-depth to use as a base for Test D##.

#### 2.2.4. Test D0#—External Patterns

To evaluate the flexibility of the trained NN, completely new inputs of different typologies are fed into the algorithm.

- Test D01 adds extra cells to the grid (5 × 5)
- Test D02 randomly fills the image with cells.
- Test D03 checks organic patterns: Voronoi, reaction-diffusion, l-system.

## 3. Results

Figures in this chapter have been exported directly from the code. Tables’ data of image comparison based on percentage similarity produced at IMGonline.com.ua (accessed on 20 December 2020) [80].

### 3.1. Training Outputs (Test A-C)

Test A01. Has proven successfully that non-direct relations between the paired images can be recognized by the cGAN (Figure 5). Few cells may appear in a wrong position.

Test A02. Immediately after the first test, there was a suspicion that shadows in grayscale could hinder the process. Figure 5 shows how results from A02 are sharper and better defined than in A01. Occlusion shadows hinder the processes because volume and ground were misunderstood by the NN. However, the image comparison algorithm did not express these improvements.

Test A03 and A04 do not specifically have any conclusion or improvement over test A01. When it comes to “understand” and rightfully express the geometry, both parts have proved equally relevant. Nonetheless, Table 5 and Figure 9 show evidence that increasing the number of echoes significantly increases the definition of the images.

**Figure 6:**
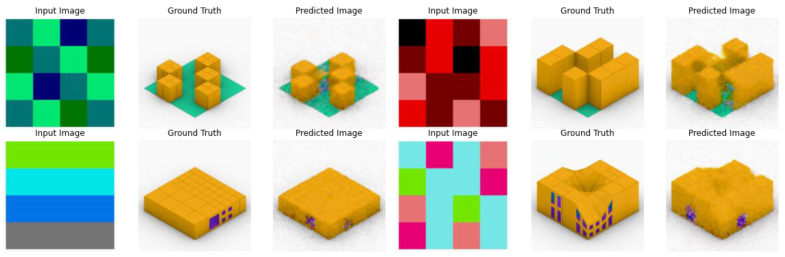
Captures from test B02. The conversion is working and the images are similar but there are mistakes in some parts that evidence that the training has not been enough.

Test B01 (Figure 6 and Table 6). As color and more complex geometry is introduced, the default training settings (250-50) proved insufficient. Although shapes could be recognizable, there is an almost complete absence of openings and interior levels, as well as some of the volumes.

Test B02 (Figure 6) shows a remarkable improvement over its predecessor. Volumes and their openings are more defined and interior colors start to appear, sometimes in the wrong order.

**Figure 7:**
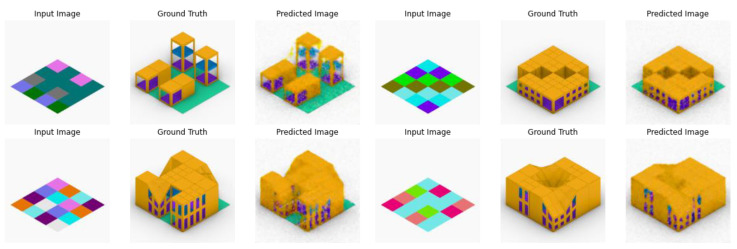
Captures from test C01 (**left**) and C02 (**right**).

Test C01. Introduced as a parallel way to compare how important the pixel distribution can be. The pattern has been converted to an isometric, so there is a lower level of abstraction. Results were better than in test B01 with the same amount of training but it brings a number of disadvantages that will be discussed in the conclusions. Colors and openings are noisy but in the right place (Figure 7).

Test C02. Thanks to the database increment, an improvement can be seen in the same way that B02 improved over B01. The results are less noisy but equally accurate.

Test C03. Over-trained when compared to its predecessors in order to check its performance in difficult situations outside of the training data base. This NN will be saved for Test D##.

### 3.2. External Outputs (Test D)

As previously described, the last group of tests has been fed with images outside of the training database. There are three subgroups described as:

(D01) Enlarging the original grid to 5 × 5 pixels.
(D02) Deconstructing the grid and spreading it through the image.
(D03) Using other patterns, like reaction-diffusion or voronoi.

**Figure 8:**
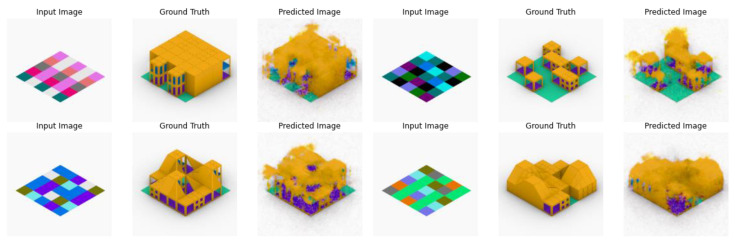
Captures from test D01.

Data from D01 is added in Table 7 and represented in Figure 8, since is the only test in group D that can be analyzed. In Figure 8 we can see how the neural network is able to intuit the position of the elements (openings, heights, colors… but the level of noise and distortion is greater than in any of the previous examples. Taking into account that the intuitions are correct, the results are extremely positive considering that a 5 × 5 pattern has never been introduced in the neural network.

**Figure 9:**
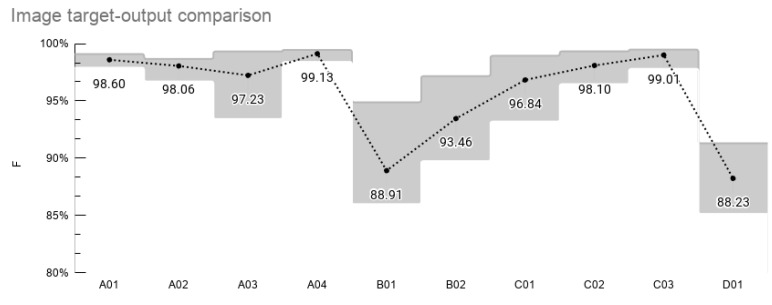
Graph comparing average (black dotted line) and min-max boundaries of each experiment (grey bars). Most relevant are: A03 due to low count of echoes, the improvement from B to C tests and the indetermination of D01.

In Figure 9, in addition to the importance of resources, we can see how test D01 is not far from the results of B01. And although it was concluded that the training in B01 was insufficient, the fact that the assessment by comparison of the images is almost the same in a network trained with examples (B01) as in one that has not been (D01) opens the door to interesting options.

While test D01 is generated parametrically (both pattern and isometric), patterns in D02 and D03 where handmade designed. For this reason, there is no possible comparison with the “right” answer.

**Figure 10:**
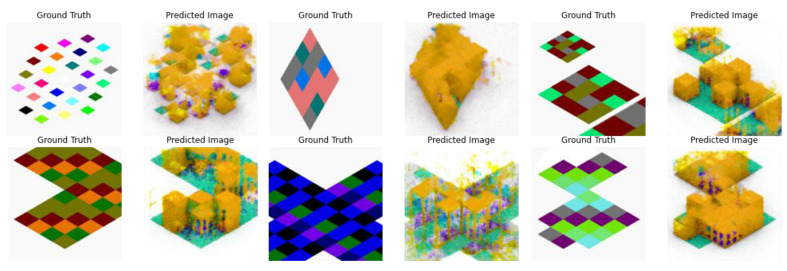
Captures from test D02.

In D02 Authors have tried to change the patterns by: separating, deconstructing, reorienting and scaling. The results are below previous experiments but still the network is capable of generating recognizable isometrics with a certain level of intuition (Figure 10). It is specially negative the resulting reoriented pattern, completely breaking the isometric representation. On the other hand, it seems that ground is better defined than roofs transitions.

**Figure 11:**
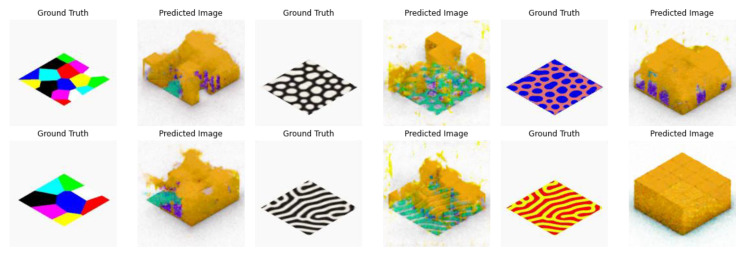
Captures from test D03.

Results in D03 proof that the NN is not prepared for geometries completely outside of its training. Although some positive things can be observed (ground treatment and color understanding), the isometric architectures fail to generate organic shapes or reinvent the original system (Figure 11).

## 4. Discussion and Conclusions

The main conclusion is that the initial hypothesis can be easily achieved by the cGAN, opening a world of possibilities around complex pattern encoding and architecture representation at many levels. The majority of tests support this claim and have shown the importance of data-base sizes and, especially the amount of echoes run. Despite the differences between pattern and isometric, every test improved as new resources where added (Figure 9). In fact, the D01 test proved to be positively intuitive despite not having received training from its inputs, practically matching the values of the B01 test that had been trained.

This approach is not only as a strategy for simplifying and compressing reality into more efficient information but also as a powerful tool to unleash complexity into architecture.

The value of the conversion goes beyond assigned specific geometries and their isometric representation with colors but the ability to understand the relation between the colors in the pattern. This fact can be clearly seen when tilted roofs appear; depending on the heights of the cells, it is a demonstration in which the cGAN establishes decisions that are outside of the color by itself and depends on neighborhood and patterns.

The authors believe that the capacity of cGANs to generate content extremely quickly in a tremendously simple process encourages their integration into the workplace. Websites or apps can run the trained GANs in the face of the difficulty of installing and learning certain software [81]. Calculating without calculating could mean a change of the paradigm in terms of simulation in fields such as structures, sunshine, energy efficiency and so forth.

In terms of graphic representation, the cGAN has successfully included omni light and occlusion shadows into the final outputs. Together with pseudo-3D representation (isometric), the results include a value that could potentially disrupt technologies like 3D modelling or computer-generated imagery.

## 5. Limitations

Some of the limitations regarding the specifics of the tests run are:

The color coding used for the generative model has a tendency toward manifesting changes in opposition to do nothing. This is due to the modifiers related to colors; since light colors are attributed to changes and the existence of the cell in the first pattern, it is most likely that those cells express the changes. In other words, although colors are split into channels, darker colors will always have a smaller frequency.

Using a fourth channel (Alpha) to create or delete cells in the grid would fix this bias but it would make visual analysis and file management more difficult (from the human point of view).

This does not affect the experiment’s success since the generative model was only a simplified version to test the conversion from color patterns into geometry through deep learning.

Besides that, the use of isometrics can sometimes be complicated by the co-incidence of certain perspectives. Reinforcing one of the axes over the others could solve this issue.

As far as the use of patterns in isometrics is concerned and despite the improvement in the behavior of the network, it is necessary to take into account what it means to establish a direct relationship between the pattern arrangement and the actual location of the architecture. Slopes and differences in the terrain would hide the color and distort the size of the pattern, making a universal conversion system unfeasible. Therefore, since the experiments in B## have been successful, it is convenient to continue investing resources in that direction. The problems mentioned -such as the slope or height of the ground- could also be solved by assigning them to specific colors or values of the pattern.

The scalability and abstraction of the patterns are not without questions. Encoding or mathematical studies associated with architecture are not without risk, as was eloquently exposed in [82]. How much can architecture be simplified? How much can we encode and abstract patterns that unfold into reality-complex architecture?

Together with resource limitations for appropriately training the NN, designers should carefully check for bias and verify the perfect functioning of their applications. As tests B01 and D01 show (Figure 9), ML’s only limitation might be the size of its database and experience, which could be overcome in subsequent years as technology improves and adapts towards AI.

In closing, it is worth commenting on the results in test D02 and D03. Most of the examples have shown a certain level of intuition but unfortunately, there is also a dramatic lack of definition. It would have been more interesting if the results had been wrong or different but well-defined. Thus, one can see that the NN does not respond well to those examples outside the training (Figure 10 and Figure 11, test D02 and D03).

It is also worth mentioning that the database used was particularly rigid in modulation and geometry and this could be the cause of the lack of response from the NN. The use of natural patterns, although interesting decisions can be seen and the grid that was turned 90°, worked particularly badly.

To conclude this research, despite the simplicity of the tests carried out, the authors recommend increasing the complexity of the projects exponentially to find its limitations. Now that the viability of the process has been demonstrated, new experiments should be carried to gain knowledge on possible levels of abstraction and how they impact on neural network learning. Similarly, the ability to scale information should also be addressed, either in the architectural/pattern design or image resolution in the neural network.

The proposed methodology can be applied to any architectural scale, providing detailed decisions to solve hundreds of encounters in a hierarchical an intelligent way, whether they are aesthetic, constructive or urban based.

Figures such as Michael Hansmeyer [83] should be considered references for their theoretical approach towards a morphogenetic architecture based on patterns and fractals. But also its application on urban design to autonomously specify smaller scale details while addressing main territory patterns or city neighborhoods [84].

## Figures and Tables

**Table 1:** Transformations based on color channel and luminance.

Channel	Transformation	Dark Color/Event	Light Color/Event
Luminance	Existence	Void	Fill
Red	Allometry	One floor	Two floors
Green	Subdivision	No subdivision	Catmull-Clark subd.
Blue	Homeobox	Solid	Openings

**Table 2:** Incrementing color levels leads to 50% void/fil ratio.

Levels	Individuals Tested	Dark Colors (Empty Cell)	Light Colors	Total Colors	Percentage of Dark Colors
2	500	411	2877	3288	12.50%
4	500	998	2282	3280	30.43%
8	500	1288	1996	3284	39.22%

**Table 3:** Possible events and their probability. Notice that the third channel value is 125 (below 127.5). Each channel position is combined in a three part color (red-green-blue).

Channel Value	Existence %	Total %	Event %
255	66% Cell creation	21.8% Light event	33.3% of events
125		44.5% Dark event	66.6% of events
0	33% Cell deletion	33.3% No event	-

**Table 4:** List of tests A to C.

Test Name	Input Image	Target Image	# Images	# Epochs	# Total Images
A01	pattern binary	iso gray	250	50	12,500
A02	pattern binary	iso red	250	50	12,500
A03	pattern binary	iso gray	500	50	25,000
A04	pattern binary	iso gray	250	100	25,000
B01	pattern color	iso color	250	50	12,500
B02	pattern color	iso color	350	70	24,500
C01	pattern iso color	iso color	250	50	12,500
C02	pattern iso color	iso color	350	70	24,500
C03	pattern iso color	iso color	500	100	50,000

**Table 5:** Image comparison data from group A.

	A01	A02	A03	A04
Average	98.60%	98.06%	97.23%	99.13%
Min. value	97.90%	96.70%	93.42%	98.41%
Max. value	99.10%	98.67%	99.33%	99.45%

**Table 6:** B-tests image comparison data.

	B01	B02
Average	88.91%	93.46%
Min. value	85.98%	89.74%
Max. value	94.88%	97.17%

**Table 7:** C## and D01 tests data.

	C01	C02	C03	D01
Average	96.84%	98.10%	99.01%	88.23%
Min. value	93.20%	96.47%	97.80%	85.13%
Max. value	98.95%	99.34%	99.48%	91.30%

## Data Availability

All images from data base are available at this public google drive folder. A folder with detailed schemes from the NN architecture also can be found. Link: https://drive.google.com/drive/folders/1KNE7K9ZWlXVlBXEtWCQLWfwHsWWYJnz3?usp=sharing.
